# A practical guide to the implementation of artificial intelligence in orthopaedic research—Part 2: A technical introduction

**DOI:** 10.1002/jeo2.12025

**Published:** 2024-05-07

**Authors:** Bálint Zsidai, Janina Kaarre, Eric Narup, Eric Hamrin Senorski, Ayoosh Pareek, Alberto Grassi, Christophe Ley, Umile Giuseppe Longo, Elmar Herbst, Michael T. Hirschmann, Sebastian Kopf, Romain Seil, Thomas Tischer, Kristian Samuelsson, Robert Feldt, ESSKA Artificial Intelligence Working Group

**Affiliations:** ^1^ Sahlgrenska Sports Medicine Center Gothenburg Sweden; ^2^ Department of Orthopaedics, Institute of Clinical Sciences, Sahlgrenska Academy University of Gothenburg Gothenburg Sweden; ^3^ Department of Orthopaedic Surgery, UPMC Freddie Fu Sports Medicine Center University of Pittsburgh Pittsburgh USA; ^4^ Department of Health and Rehabilitation, Institute of Neuroscience and Physiology, Sahlgrenska Academy University of Gothenburg Gothenburg Sweden; ^5^ Sportrehab Sports Medicine Clinic Gothenburg Sweden; ^6^ Sports and Shoulder Service, Hospital for Special Surgery New York New York USA; ^7^ IIa Clinica Ortopedica e Traumatologica, IRCCS Istituto Ortopedico Rizzoli Bologna Italy; ^8^ Department of Mathematics University of Luxembourg Esch‐sur‐Alzette Luxembourg; ^9^ Fondazione Policlinico Universitario Campus Bio‐Medico Rome Italy; ^10^ Research Unit of Orthopaedic and Trauma Surgery, Department of Medicine and Surgery Università Campus Bio‐Medico di Roma Rome Italy; ^11^ Department of Trauma, Hand and Reconstructive Surgery University Hospital Münster Münster Germany; ^12^ Department of Orthopedic Surgery and Traumatology, Head Knee Surgery and DKF Head of Research Kantonsspital Baselland Bruderholz Switzerland; ^13^ Center of Orthopaedics and Traumatology University Hospital Brandenburg a.d.H., Brandenburg Medical School Theodor Fontane Brandenburg a.d.H. Germany; ^14^ Faculty of Health Sciences Brandenburg Brandenburg Medical School Theodor Fontane Brandenburg a.d.H. Germany; ^15^ Department of Orthopaedic Surgery Luxembourg Centre Hospitalier de Luxembourg—Clinique d'Eich Luxembourg Luxembourg; ^16^ Luxembourg Institute of Research in Orthopaedics Sports Medicine and Science (LIROMS) Luxembourg Luxembourg; ^17^ Luxembourg Institute of Health, Human Motion, Orthopaedics Sports Medicine and Digital Methods (HOSD) Luxembourg Luxembourg; ^18^ Clinic for Orthopaedics and Trauma Surgery Erlangen Germany; ^19^ Department of Orthopaedics Sahlgrenska University Hospital Mölndal Sweden; ^20^ Department of Computer Science and Engineering Chalmers University of Technology Gothenburg Sweden

**Keywords:** artificial intelligence, machine learning, orthopaedics, research methods, sports medicine

## Abstract

Recent advances in artificial intelligence (AI) present a broad range of possibilities in medical research. However, orthopaedic researchers aiming to participate in research projects implementing AI‐based techniques require a sound understanding of the technical fundamentals of this rapidly developing field. Initial sections of this technical primer provide an overview of the general and the more detailed taxonomy of AI methods. Researchers are presented with the technical basics of the most frequently performed machine learning (ML) tasks, such as classification, regression, clustering and dimensionality reduction. Additionally, the spectrum of supervision in ML including the domains of supervised, unsupervised, semisupervised and self‐supervised learning will be explored. Recent advances in neural networks (NNs) and deep learning (DL) architectures have rendered them essential tools for the analysis of complex medical data, which warrants a rudimentary technical introduction to orthopaedic researchers. Furthermore, the capability of natural language processing (NLP) to interpret patterns in human language is discussed and may offer several potential applications in medical text classification, patient sentiment analysis and clinical decision support. The technical discussion concludes with the transformative potential of generative AI and large language models (LLMs) on AI research. Consequently, this second article of the series aims to equip orthopaedic researchers with the fundamental technical knowledge required to engage in interdisciplinary collaboration in AI‐driven orthopaedic research.

**Level of Evidence:**

Level IV.

**Abbreviations**
ACL: anterior cruciate ligament
AGI: artificial general intelligence
AI: artificial intelligence
BERT: Bidirectional Encoder Representations from Transformers
CNN: convolutional neural networks
CT: computerised tomography
DL: deep learning
GAN: generative adversarial networks
GMAI: generalist medical AI
GPT: generative pretrained transformer
LLaMA: Large Language Model Meta AI
LLMs: large language models
LSTM: long short‐term memory
ML: machine learning
NLP: natural language processing
NN: neural networks
PaLM: Pathways Language Model
PCA: principal component analysis
PROs: patient‐reported outcome measures
RL: reinforcement learning
RNN: recurrent neural network
SVM: support vector machine

## INTRODUCTION

Advances in computing power, multimodal data and unprecedented scientific applications of artificial intelligence (AI) in medicine present a broad range of possibilities across the field of orthopaedics. Orthopaedic domain knowledge and clinical research methods are essential components in the design of studies that yield high‐quality clinical evidence. However, fundamental technical literacy in AI is currently a rate‐limiting step for the successful implementation of AI‐driven scientific discovery and clinical applications in orthopaedics. The aim of this article is to familiarise orthopaedic researchers with the rudimentary technical knowledge required to conceptualise how AI algorithms work and the types of problems they are suitable for solving. Specifically, we will focus on the subfield of machine learning (ML), which has been the main driver behind numerous advances in AI over the recent years. The key characteristic is that ML enables computers to learn from and make decisions based on data, without being explicitly programmed for specific tasks. In ML, algorithms analyse patterns in large data sets, such as medical images or patient records to make predictions or identify trends.

## KEY TECHNICAL TERMS FOR GETTING STARTED WITH AI‐DRIVEN RESEARCH

AI refers to a field of computer science focussing on the development of systems for performing tasks that typically require human input in terms of behaviour and decision‐making. In general, such tasks involve recognising and understanding patterns, understanding and interpreting natural language, predicting future events and complex, domain‐specific problem solving. The continuously evolving landscape of AI and ML leads to considerable variability in the categorisation and terminology used when discussing AI. Nevertheless, a basic theoretical understanding of AI can be achieved based on the capabilities of a given AI system: [55]

1. *Narrow AI* is the only form of AI that is currently implemented and applied in several disciplines within and outside of the medical domain. Designed to perform a specific task, narrow AI systems have consistently shown the ability to augment the performance of human clinicians in those specific domains [9, 54, 71, 72, 76]. However, narrow AI systems remain limited to performing an assigned task and are unable to perform well outside of the predefined framework. In the context of orthopaedic research, narrow AI systems have been put to the test for carrying out tasks like fracture detection and classification based on radiographic images [8, 39, 51] and disease [88], injury risk [36] and surgical outcome prediction [37, 38, 56], with impressive domain‐specific capabilities.
2. *Artificial general intelligence (AGI)* is the hypothetical capability of AI to adapt to new tasks in various contexts without human oversight. While this level of adaptability without human intervention is theoretically powerful for solving various challenges in medical research and the clinical setting, AGI remains a theoretical concept upon the publication of this text in 2024. While likely several years away, it is reasonable to expect that current narrow AI systems will gradually acquire more and more general capabilities and thus approach more general and adaptive behaviour, suitable for a broad range of tasks. It is useful to view these as a spectrum from more narrowly framed tools to more generally applicable and adaptive solutions.
3. *Superhuman AI* is a theoretical construct that involves the endowment of an AI system with cognitive reasoning and emotional abilities superior to those of humans, which in turn would give way to independent motivations, beliefs and actions of the system. While such systems are not likely to be built in the near future, some computers already possess the ability to perform several tasks with superhuman proficiency, for example, calculations and rapid summary of long documents, with consideration for millions of possible scenarios. It can thus be expected that even narrow AI systems will demonstrate superhuman performance in certain aspects of function or problem‐solving capability. An example of this is the possibility to identify and categorise information based on patterns in several million patient health records.

Accordingly, the current learning series will focus on the application of narrow AI (henceforth referred to as AI) systems to learn from data and optimise their behaviour over time, which promises to be particularly powerful when used for research in health care. However, it is important to acknowledge that such models will gradually become more general and adaptive and exhibit certain superhuman characteristics. Orthopaedic researchers aiming to use AI in their research projects are encouraged to familiarise themselves with the complex taxonomy of AI, underlying principles and properties at each hierarchical level, along with their possible applications across the orthopaedic research landscape.

## UNDERSTANDING THE TOOLBOX OF METHODS FOR AI‐DRIVEN RESEARCH

**Figure 1:**
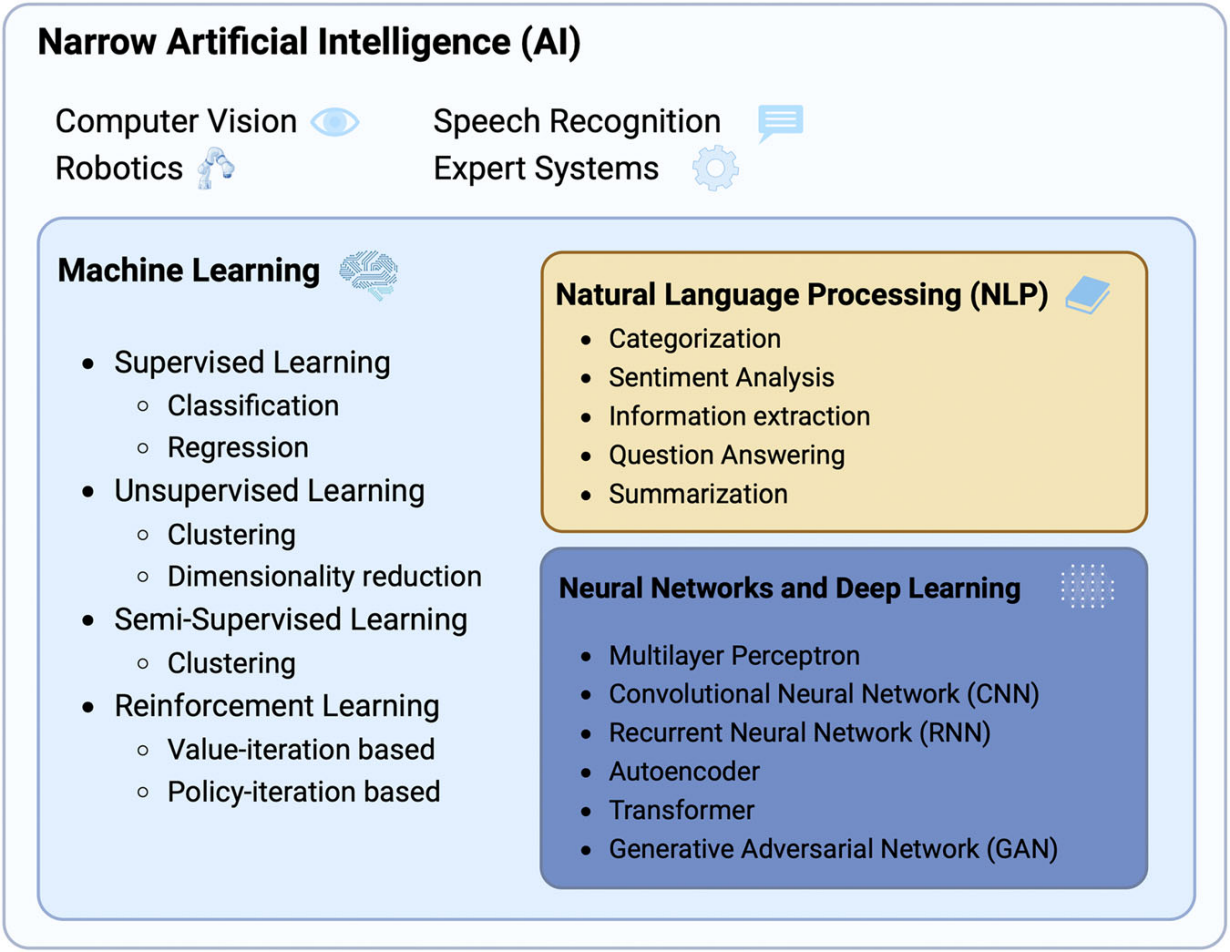
The diagram illustrates the subdomains of narrow artificial intelligence (AI), including levels of supervision and the most frequently applied methods according to each subdomain.

The aim of the following section is to present a systematic and holistic perspective of AI and the associated subcategories of methods referred to when discussing the use of AI for biomedical research (Figure 1). While computer vision, speech recognition, robotics and expert systems are broad subdomains of AI in their own right, the present discussion will be limited to the description of computational techniques suitable for clinical research in orthopaedics without prerequisites in engineering disciplines [66]. Orthopaedic researchers wishing to delve deeper into the technical workings of specific models and the interpretation of their outputs are referred to additional literature on the subject [16, 33, 40, 49, 50, 58, 59].

### ML

ML is probably the most widely used form of AI in medical research with clinical translation [6]. In broad terms, ML aims to replicate the human ability to recognise objective patterns based on inherent characteristics of a data set using computational methods. Typically, a set of layered mathematical algorithms or formulas of a given ML system is used to represent (more commonly referred to as ‘model’) scientific phenomena based on patterns learned from the data set that was used for training the system. Depending on the given research problem, the type of ML model and the characteristics of the data set, the ML model may then be applied to new, previously unseen data to perform tasks such as classification, detection, cluster analysis and regression based on the associations learned by the model. The ability of ML models to characterise relationships encoded in large and diverse data sets is particularly useful in diagnostic and clinical decision‐making scenarios that provide cognitive challenges to humans both in terms of complexity, the number of data points to be considered and the limitations posed by cognitive biases that lead to human error. Consequently, the increasing volume of multimodal biomedical data available for academic research are abundant in features that render them suitable for solving research problems in a reproducible and time‐efficient manner with ML approaches. However, it is important to note that while ML methods are useful to identify associations and correlations within input variables and a certain outcome, these are not equivalent to cause‐and‐effect relationships and should be used cautiously for inferential clinical reasoning. While research in the domain of causal ML is not sufficiently mature to cover in this introductory article, it is expected to play a crucial role in the future development of interpretable and actionable clinical AI systems [70].

In response to an input (previously unseen data), ML models respond with numeric, discrete, categorical or probability‐based outputs based on relationships within the labelled or unlabelled data the given model was trained on. However, ML models vary in terms of the degree of required human oversight, model‐specific characteristics and inherent mathematical layers implemented for data analysis and learning. A fundamental understanding of such specifications is essential to orthopaedic researchers for proficiency in task‐specific model selection and the successful design of AI‐driven research projects.

### The spectrum of supervision in ML

**Figure 2:**
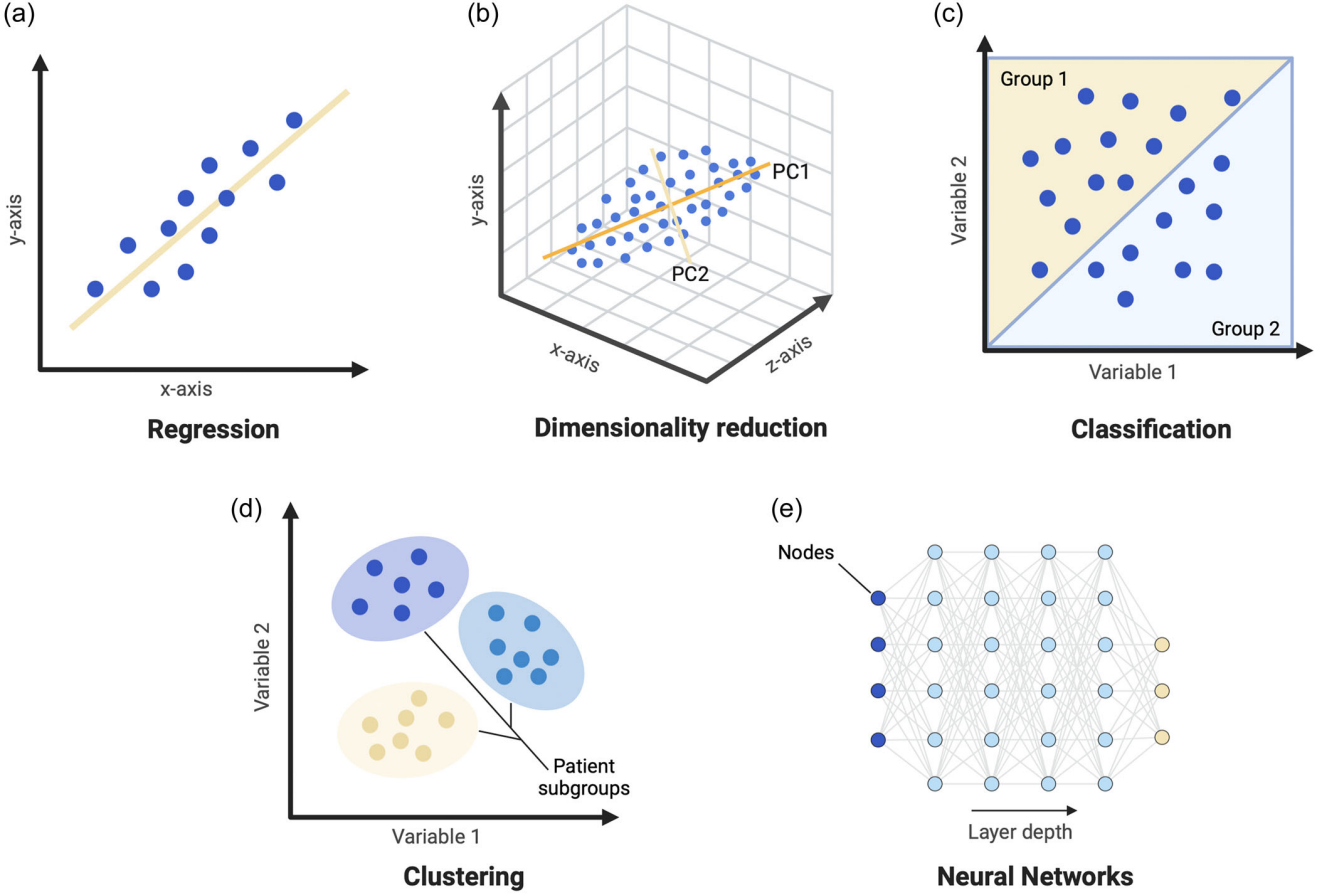
A schematic representation of commonly performed machine learning tasks. (a) Regression: a line (yellow) providing the best fit to the data (blue dots) is applied and the model can be used to predict a continuous outcome (y) based on one or several predictor variables (x). (b) Dimensionality reduction: enables a reduction in the number of variables considered for modeling an outcome through feature selection and/or extraction. This is illustrated by reducing a three‐dimensional data set (blue dots) into two principal components (yellow lines: PC1 and PC2) through principal component analysis (PCA). (c) Classification methods are used to assign data points (blue dots) into two or more classes (yellow and blue triangles) based on differences in characteristics, which the model can interpret as boundaries to separate data. (d) Clustering involves the separation of input data into two or more clusters based on similarities and differences in a set of characteristics. The illustration displays three patient subgroups (yellow, blue and purple ovals) identified within a hypothetical data set (blue dots) using a clustering approach. (e) Neural networks are organised in layers of algorithms that mimic the interconnectedness of neurons in the brain. The illustration displays a neural network with interconnected nodes arranged in multiple connected layers of a certain depth. Data at the input level (dark blue node) are transmitted through subsequent layers of the network (light blue nodes) until the layer providing the output (yellow nodes) is reached.

To develop models for predicting a certain outcome based on new data, an ML model requires access to ‘ground truths’ acquired either when the training data set was collected or added when the model was to be fitted to the training data set. Supervised ML refers to the inherent possession or newly defined ground truths for a model through manual identification (also referred to as labelling) of the input and output variables in the training data, typically performed by humans with domain expertise within the area of research or based on objective measurements from reliable instruments (e.g., the prediction of ACL revision surgery risk based on quantified anteroposterior and rotatory knee laxity measured with validated devices [46]). As a result, supervised ML models learn patterns and associations between components of the training data set deemed relevant to human labellers and the manually labelled or objectively determined outputs (Table 1). Examples of supervised ML approaches in orthopaedic research include outcome prediction following arthroscopic treatment of femoroacetabular impingement surgery [48] and the prediction of anterior cruciate ligament (ACL) reconstruction revision risk using national registry data [41]. Manual labelling is both a time‐consuming and labour‐intensive process, which is often disadvantageous in a clinical research setting. Unsupervised ML bypasses human input through automated pattern detection in unlabelled data. Consequently, unsupervised ML removes the constraint of human bias introduced through manually assigned labels and may elucidate more complex, implicit relationships within data sets, which may be actionable but also challenging to interpret. Applications of unsupervised ML approaches have shown excellent results in classification and clustering tasks, particularly useful in the identification of clinically relevant patient subgroups. Examples in orthopaedic research include the detection of patient phenogroups in osteoarthritis based on clustering analysis of biomarker data [4] and the stratification of total hip arthroplasty patients into clinically meaningful, risk‐based subgroups [37]. Rather than a choice between one method or the other, supervised and unsupervised ML exists on a spectrum. Semisupervised learning [81] makes use of both labelled and unlabelled data to train a model and make subsequent predictions. In contrast, self‐supervised learning [62] is implemented through partial manual labelling of the available data, followed by automated prediction of the remaining labels through unsupervised methods. Both methods aim to combine the advantages of supervised and unsupervised approaches to ML. It is also important to mention reinforcement [34] and transfer learning [22]. Reinforcement learning (RL) refers to methods that enforce training models with the help of positive and negative feedback with a trial‐and‐error approach for model fitting [68]. In transfer learning, pre‐existing models trained for specific tasks are used to enhance the performance of a new model trained for a different task, where knowledge gained from previous models allows for improved performance and a reduced amount of data required for training the new model [22]. Examples of previous research using reinforcement and transfer learning in medicine include decision support tools for the treatment of sepsis [87] and the optimisation of automated medical image analysis [2, 29, 90]. The next section will focus on introducing the conceptual basics of the technology behind frequently used ML algorithms across the spectrum of supervised and unsupervised learning for tasks like classification, clustering, regression, dimensionality reduction, neural networks (NNs) and deep learning (DL) (Figure 2). It is important to mention that models based on deep NN architectures have over the recent years displayed superior performance in classification, clustering, regression and dimensionality reduction tasks and will be discussed separately in further detail [67].

**Table 1:** A glossary of essential concepts and terms for AI‐driven research.

Supervised learning	A machine learning approach where models are trained on labelled data (either by human labelers or from a trusted, objective measurement) to make predictions or classifications based on input data.
Unsupervised learning	A machine learning approach where models identify patterns and relationships in data without the use of labelled outputs.
Semisupervised learning	A paradigm that falls between supervised learning and unsupervised learning, beneficial in settings of resource‐intensive data acquisition and when unlabelled data may help enhance model performance and generalisability.
Reinforcement learning	A machine learning paradigm where agents learn to make decisions by taking actions in an environment and receiving feedback in the form of rewards or penalties.
Self‐supervised learning	A type of unsupervised learning where models generate labels from the data themselves, often by predicting parts of the input data from other parts.
Ensemble learning	A machine learning technique that combines multiple models to improve prediction accuracy and reduce overfitting.
Transfer learning	A method where a model trained on one task is leveraged for a related task, reducing the need for extensive data and training time.
Deep learning	A subfield of machine learning that utilises neural networks with multiple layers to automatically learn and extract features from data, often used for tasks like image and speech recognition.
Data augmentation	Techniques for expanding training data sets by creating new data points from existing data, improving model performance.
Model interpretability	The ability to understand and explain how a machine learning model arrives at specific decisions or predictions, ensuring transparency in the model's decision‐making process.
Model explainability	The ability to provide a clear, understandable and often human‐readable explanation for the decisions and predictions made by a machine learning model.
Classification	A type of machine learning task where the goal is to assign data points to predefined categories or classes based on their features.
Regression	A machine learning task aimed at predicting a continuous numeric value, often used for tasks like forecasting a quantitatively measured outcome.
Clustering	An unsupervised learning task where data are grouped into clusters based on similarity or proximity.
Labelling	The process of assigning categorical labels or values to data instances, a crucial step in supervised learning.
Parameters and hyperparameters	In machine learning, parameters are the internal settings or variables learned by a model during training, while hyperparameters are external settings that govern the learning process, such as learning rates and model architecture.
Underfitting	Occurs when a machine learning model is too simple to capture the underlying patterns in the data, resulting in poor performance on both training and testing data sets.
Overfitting	Occurs when a machine learning model is overly complex and fits the training data too closely, resulting in poor generalisation to new, unseen data.
Training	The process of teaching a machine learning model by providing it with labelled data and iteratively adjusting model parameters to minimise prediction errors.
Testing	The evaluation process where a trained machine learning model's performance is assessed using an independent data set to estimate its generalisation capabilities.
Validation	A separate data set used during model training to tune hyperparameters and assess model performance, helping to avoid overfitting.
Inductive bias	The inherent assumptions or prior knowledge incorporated into a machine learning model to facilitate learning and decision‐making.
Dimensionality reduction	The process of reducing the number of features or dimensions in data, often to enhance model performance, visualisation or efficiency.
Distributional shift	A change in the underlying data distribution, which can occur between the training and testing data sets and impact model performance in real‐world applications.
Black box decision‐making	Refers to decision‐making processes in machine learning models that are not easily understandable or explainable due to complex internal workings.
White box decision‐making	The opposite of black box decision‐making, where machine learning models produce results that are transparent, interpretable and can be explained using clear rules and logic.

Abbreviation: AI, artificial intelligence.

### Classification

The objective of classification in ML is to determine the category to which new data points belong based on predictive modelling of the training data. Classification tasks can be binary (one of two), multiclass (one of many) or multilabel (several of many) depending on the number of classes and the hierarchical structure of classes within a given data set. Performing classification with ML lends itself well to both structured (typically organised in relational databases or tables) and unstructured (unorganised) data. The method typically involves the mapping of mathematical functions with inherent assumptions to identify boundaries between distinct output classes (y) based on certain features of the labelled or unlabelled input variables (x). Popular classification algorithms range from logistic regression, linear discriminant analysis, naive Bayes [85], K‐nearest neighbours [78], support vector machine [5], decision tree [30], random forest [68], gradient boosting [7] and rule‐based classification [68] algorithms to deep NNs [67] (Table 2).

**Table 2:** A general overview of AI methods and their applicability to specific types of orthopaedic research questions based on the data type required and specific model characteristics.

Method	Example of application	Required data type	Specific characteristics
Convolutional neural network (CNN)	Fracture detection based on radiographic imaging	Radiographs with labelled fracture locations	Appropriate technique for image analysis tasks
Deep neural networks	Predicting patient recovery time following total knee joint arthroplasty	Patient records, including demographic, surgical and postoperative data	Versatile but may require substantial quantities of data and computational power
Generative adversarial networks (GANs)	Generating synthetic 3D models of the musculoskeletal system for simulations	Computerised tomography (CT) data for information regarding 3D bone structure with labels for training	Useful for synthetic data generation and data augmentation
Gradient boosting	Identifying optimal implant placement in orthopaedic surgery	3D models of bone structures and implant specifications	Powerful for regression tasks and ensemble learning
K‐nearest neighbour (K‐NN)	Predicting the risk of ACL reinjury based on the intensity level of sporting activity performed after surgery	Patient demographic data (age, gender) and Tegner activity level	Suitable for similarity‐based tasks
Long short‐term memory (LSTM) networks	Predicting recovery trajectory after ACL reconstruction based on time‐series patient data	Time‐series patient data including patient‐reported outcome measures (PROs), muscle function and psychological risk appraisal	Data should be sequential with temporal dependencies
Principal component analysis (PCA)	Reducing the dimensionality of feature sets for orthopaedic data analysis	Multidimensional orthopaedic data including text, patient‐reported outcome measures and radiologic imaging	Suitable for the simplification of complex data sets through feature extraction
Random forest	Predicting the likelihood of surgical complications	Patient records, including medical history and surgical data	Effective for high‐dimensional data and complex relationships
Recurrent neural network (RNN)	Predicting the progression of musculoskeletal disorders over time	Time‐series data on patient symptoms and treatment history	Suited for sequence modelling in longitudinal studies
Support vector machines (SVMs)	Classifying bone fractures based on radiographic images	Labelled medical images (radiographs) and diagnostic data	Effective for binary classification tasks
Transformers	Analysing patient notes and radiology reports for diagnosis	Text data such as clinical notes, medical reports and radiology findings	Suitable for processing sequential data
Autoencoders	Reducing dimensionality in bone density data for visualisation	Bone density measurements and corresponding spatial data	Dimensionality reduction and feature extraction
Bayesian networks	Assessing the probability of sustaining orthopaedic injuries based on the type of sporting activity performed	Demographic and injury‐related variables	Excels with probabilistic modelling

Abbreviation: AI, artificial intelligence.

### Regression

**Figure 3:**
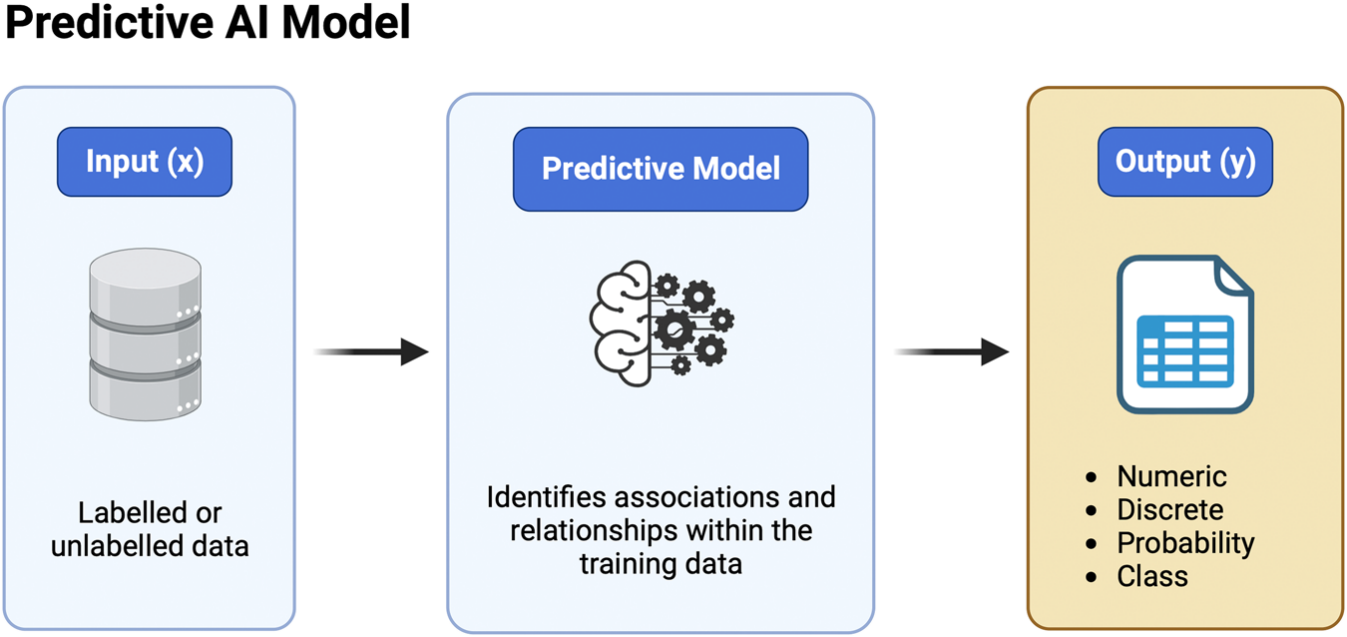
The diagram displays the basic components of predictive artificial intelligence (AI) models, including labelled and unlabelled data at the input level (x) and numeric, discrete, probability‐based or class‐based variables at the output level (y).

In contrast to classification, which predicts distinct class labels, regression analysis with ML enables the prediction of outcomes measured on continuous numeric scales (Figure 3). Mathematically, a function is mapped to a data set to model the linear or nonlinear relationship between one or several predictor variables (x) and a continuous outcome label (y) [68]. Regression models lend themselves particularly well to modelling and forecasting responses to medical interventions in terms of subjective and objective outcome measures reported on continuous scales. Frequently used examples of regression algorithms in AI‐driven medical research include simple and multiple linear regression [68], gradient boosting [7], polynomial regression [68], decision tree and random forest‐based approaches [30, 66], least absolute shrinkage and selection operator (LASSO) and ridge regression [20] and deep NNs [67] (Table 2).

### Clustering

Clustering is an unsupervised or semisupervised ML approach for dividing data into distinct groups (clusters) based on the distribution of identified trends within the various dimensions of the given data set [16]. Cluster analysis can be performed using partitioning methods to separate data based on similarities and differences in terms of relevant features, density‐based methods with a focus on the spatial distribution of data, hierarchy‐based and grid‐based methods where clusters are identified at various layers of complexity within the data set, model‐based methods that use statistical methods or NNs and constraint‐based methods that incorporate domain knowledge [68]. Commonly used clustering algorithms (Table 2) include K‐means clustering [25] (distribution based), agglomerative hierarchical clustering [52] (hierarchy based), density‐based spatial clustering of applications with noise [65] (density based), Gaussian mixed model clustering [84] (distribution based) and deep NN architectures [67].

### Dimensionality reduction

While multimodal data sets consisting of a large number of variables are required for the analysis of complex relationships within medical data, making sense of this complexity may also present challenges in terms of computational costs and human interpretability [16]. Through an unsupervised approach, dimensionality reduction enables the simplified analysis of complex data sets through the elimination of unimportant data, while maintaining data that are salient for modelling an outcome. Dimensionality reduction can be achieved by means of feature selection or feature extraction [68]. Feature selection involves the selection of a subset of variables from the original data for an analysis with lower dimensionality, while feature extraction relies on the creation of new features that reflect interactions among several variables from the original data set, while retaining the essential information [68]. Frequently used methods of dimensionality reduction include principal component analysis [57], recursive feature elimination [53], linear discriminant analysis [89] and autoencoders [43], among others.

### RL

The method of RL involves training of an interactive agent to take desired actions within a predefined context. In response to actions taken within the defined environment, agents may subsequently learn to take actions to maximise a cumulative reward, which results in learning an optimal strategy based on the provided feedback. Notably, RL has been applied to solve problems in the domains of game theory, robotics and the optimisation of complex systems and processes in medicine, manufacturing and logistics, complementing other frequently used ML methods like supervised and unsupervised learning. Applying RL to solve real‐world problems requires defining four components, specifically an agent, environment, policy and reward. While both model‐based and model‐free approaches to RL exist, model‐free approaches are advantageous in the complex environments encountered in medical research, as they provide simplicity and robustness, computational efficiency and transferability across various tasks. However, the choice between model‐based and model‐free methods depends on the characteristics of the given problem and the available data. Frequently used RL methods include Monte Carlo techniques [63], Q‐learning [24], deep Q networks [34], probabilistic inference for learning control [13] and additional hybrid approaches [68].

### NNs and DL

NNs and DL are subfields of ML inspired by the architecture and function of neurons in the human brain and have gained substantial attention in scientific research due to their excellent ability to accurately model processes and systems [67, 68]. NN models consist of functions that can be considered as artificial neurons, which are grouped into layers within the model. The first layer of the model accepts input variables from a given data set, which are processed by the functions of this first layer. The outputs of the first layer are then propagated to a new group of functions at the next layer of the model, and this process is repeated based on the number of layers in the model, also known as the depth of the NN. The final layer provides the final network model output, which may be a classification, regression or clustering output, depending on the assigned task. DL, also referred to as deep neural networks (DNNs), indicates the presence of a large number of internal layers of the model [67]. The nodes or artificial neurons of the network layers can be arranged in various configurations, resulting in a broad array of network architecture types applicable to medical research problems. More advanced architectures can also transmit feedback from intermediate results or predictions to the initial layer to enable the processing of sequential data [67]. Connections between layers of the models can be assigned different weights, modifying the importance of the individual nodes to the overall model. These weights are then updated throughout the training process of the model. While several training methods can be employed, the most frequently used method is termed backpropagation [67]. Multilayer perceptrons [77] are the simplest examples of NNs and consist of network layers arranged in a feedforward linear fashion, suitable for classification and regression tasks. More sophisticated methods, such as convolutional neural networks (CNNs) [15] are especially suitable for the analysis of data with spatial dimensions, including medical images and a video [35]. At a fundamental level, CNNs employ square‐shaped matrices called convolutional kernels or filters, which ‘slide’ or convolve across the input data (e.g., a medical image), while recognising and capturing local patterns in the data (such as sharp edges, changes in colour intensity or texture, etc.) [15]. This approach allows models to learn important features of the input data. In contrast, data structured in an ordered sequence such as time series and natural language are more appropriately processed with recurrent neural networks (RNNs) [69]. Models based on RNNs are best thought of as blocks of NN layers, which are interconnected in cycles to maintain the memory of previously entered and processed data. Autoencoders [43] are NNs designed for unsupervised tasks that involve learning compressed representations of the input data, a process also known as encoding. Subsequently, the input data can be reconstructed from the compressed representation, which is a process termed decoding. The utility of autoencoders lies in the process of feature representation, which enables the extraction of valuable information from the input data to solve dimensionality reduction, generative modelling and model fine‐tuning problems, to name a few. In contrast, transformers [1, 82] are NNs typically trained in a supervised manner, which process and learn context from sequences of tokenised information, like words, subwords or even subimages when used for imaging tasks. In this setup, the encoder creates context‐specific representations for each token (embeddings), while forming a distinct embedding for the entire sequence. A decoder is then used to convert the encoder output and thereby generate token sequences as a final output. Transformer models have gained increasing attention since their use in the development of popular language models like the Bidirectional Encoder Representations from Transformers (BERTs) [14] and generative pretrained transformer (GPT) 3 and 4 models [10]. Transformers possess built‐in attention mechanisms that enable models to adaptively focus on different aspects of the input data when making predictions about the output to be generated [80]. Autoencoders and transformers are suitable for different purposes and have revolutionised the field of DL. While autoencoders are geared towards learning compact representations and reconstruction within data, transformers excel at the efficient processing and understanding of sequential, multimodal data. Finally, it is important to mention generative adversarial networks (GANs) [12, 18], which have played an instrumental role in the development of generative tasks performed with AI. The central tenet of GANs is an adversarial training process that involves a generator and discriminator component, which engage in a continuous game with one another [12]. The generator layer is tasked with the creation of synthetic data with a distribution that is indistinguishable of the training data, while the discriminator layer detects the probability of the synthetic data originating from the generator, rather than the original data set. Feedback from the discriminator is used to improve the ability of the generator to create indistinguishable synthetic data, and this iterative process results in the improvement of both the generator and discriminator over cycles, which results in the refinement of the quality of the generated data [18]. The architecture of GANs can in turn be harnessed to create synthetic data and images [27]. In orthopaedic research, this method may be particularly useful for the augmentation of incomplete data sets with synthetic imaging, qualitative or quantitative data [23, 27, 75].

It is important to note that this survey is nonexhaustive and that a large number of additional architectures and hybrid approaches exist (e.g., GAN‐style training of models with transformer components). Given the recently reported positive results and increased interest in DNNs, the constant evolution of new architectures and training methods is likely to continue for years to come.

## NATURAL LANGUAGE PROCESSING (NLP)

NLP is an AI technique that enables machines to understand and generate natural language [28, 68]. Natural language understanding is achieved through the extraction of linguistic entities, emotions and relevant concepts from various forms of language [28]. In contrast, natural language generation is accomplished through the generation of short or long fragments of written or spoken language based on a digital representation of the linguistic and informational content of the given language [28]. Importantly, the scope of NLP is not only restricted to the structural aspects of language like sentences, words and syntax but also takes into account context, semantics, emotional content, tone and meaning. Potential applications of NLP in medical research include text classification, content extraction, question answering and decision support, sentiment analysis and summarisation tasks, which may facilitate the management and understanding of orthopaedic research data stored in the form of structured and unstructured text and expedite existing clinical documentation practices [60, 91, 92]. Popular models used for NLP tasks in research include hidden Markov [3], conditional random fields, support vector machine [17], naive Bayes [85], word embedding [44] and long short‐term memory [21] models. In the recent years, advances in NN and transformer model architectures have skyrocketed the implementation of NLP use cases through BERT [14] and GPT [10] foundation models, leading to new frontiers in AI‐driven research with generative applications.

## GENERATIVE AI AND LARGE LANGUAGE MODELS (LLMs)

**Figure 4:**
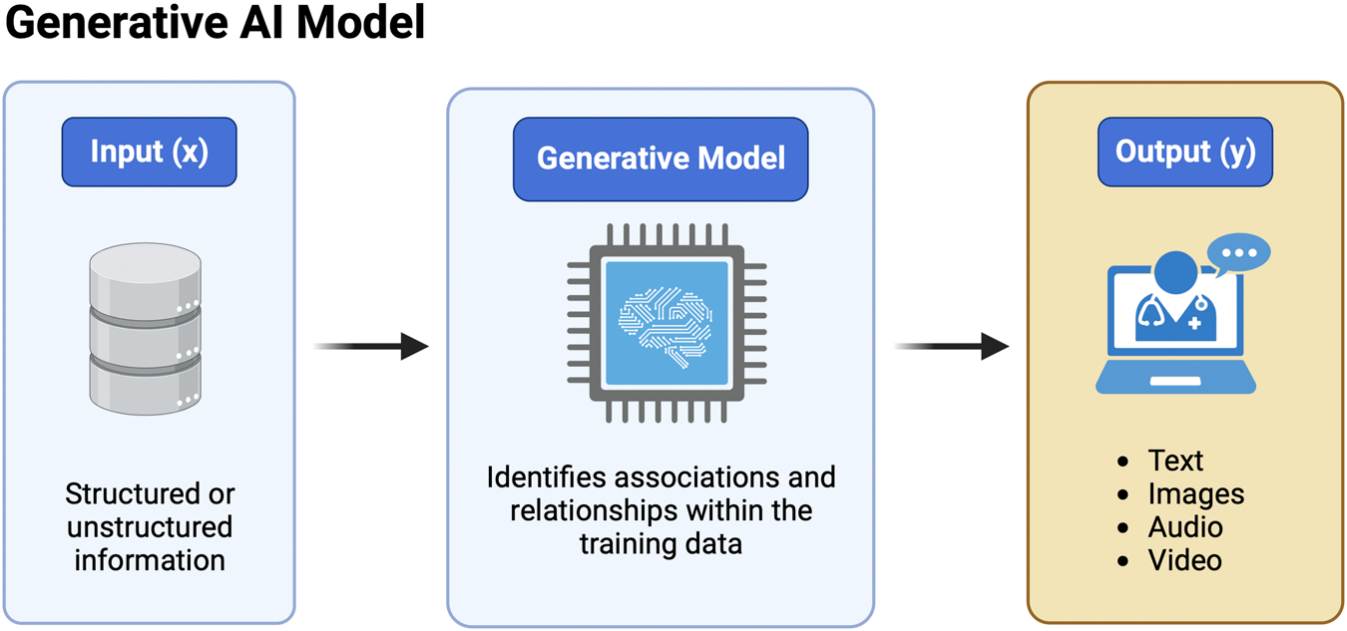
The diagram displays the basic components of generative artificial intelligence (AI) models, which accept structured or unstructured data as input (x) and return text, images, audio, video or other generated content as output (y).

Recent advances in DL techniques, transformer architectures, computing power and the scale of available data for model training have catalysed the transformation of AI research through generative AI [19, 45]. Generative AI is a branch of AI related to models with the ability to synthesise new digital content when pretrained on diverse labelled and unlabelled data sets [45]. In turn, generative AI models respond to a given input by generating output in the form of natural language, images, audio or other media types based on patterns learned from the informational content of the training data (Figure 4). Foundation models for generative AI can typically be trained on a vast array of data including text, images, video, computer code and audio, and can generate new content of the same or, in more recent use cases, different format, as the input source through conversational interaction [45]. Importantly, foundation models can be fine‐tuned through further training on more specific data (e.g., clinical notes, consensus documents, research publications, etc.) to suit a broad range of applications [91]. Additionally, more contemporary LLMs possess the ability to generate data of various modalities with little to no pretraining or fine‐tuning in a specific knowledge domain [11, 47]. While the creativity and diversity of generative AI applications are seemingly boundless, there are currently relatively few use cases documented in the orthopaedic medical literature [26]. The ability of LLMs to understand and generate human language in the form of text and audio have gained particular attention at the intersection of AI and medicine [32, 42]. One recent study determined that the GPT‐4 model generates human‐level question answering capabilities in the domain‐specific context of ACL injury and treatment [26]. At the time of this writing in 2024, popular foundation models for generative AI include large language and image generation models like BERT [14], GPT [10], Pathways Language Model (PaLM) [73, 74], Large Language Model Meta AI (LLaMA) [79], Claude 2 (Anthropic PBC) [86], Stable Diffusion [64] and DALL‐E [61]. Recent advances in generative AI led to the proposal of multimodal, generalist medical AI (GMAI) models, capable of complex reasoning and decision‐making in clinical scenarios [45]. While these models are promising for the future integration of AI in everyday medical practice, such foundation models rely on meticulously curated and annotated multimodal domain knowledge across the broad range of medical specialties and subspecialties, including orthopaedics.

## CAN AI ENHANCE SCIENTIFIC UNDERSTANDING AND DISCOVERY IN ORTHOPAEDICS?

As illustrated by the current review of the taxonomy of AI, the advancement of AI models has provided the means to digitally replace aspects of human intelligence essential for scientific understanding, including perception, reasoning, learning, complex problem solving and linguistic expression. It is therefore natural that the following question arises: how can AI‐driven research enhance scientific understanding in orthopaedics? Furthermore, how can we interpret the results of AI models and make sense of the logic used to identify hidden associations and patterns in complex multimodal medical data? It is likely that AI‐driven approaches can enhance both inductive and deductive reasoning in orthopaedic research, expand scientific understanding based on existing premises and assist with the generation of new hypotheses [31, 83]. The next section of this learning series will aim to expand on this topic and highlight ways orthopaedics research may benefit from the implementation of AI‐based approaches.

## CONCLUSION

The current article presents a comprehensive but nonexhaustive review of the fundamental technical background of AI and the taxonomy of relevant subfields for medical research applications. While a deeper technical understanding, which is facilitated by interdisciplinary collaboration, is required for the successful implementation of AI‐driven research endeavours in orthopaedics, the aim of this introductory text is to provide a basic understanding of AI to orthopaedic researchers to efficiently communicate ideas and plan in the context of an interdisciplinary research environment.

## AUTHOR CONTRIBUTIONS

Review of the literature and primary manuscript preparation were performed by Bálint Zsidai, Janina Kaarre, Eric Narup and Robert Feldt. Editing and final manuscript preparation was performed by Bálint Zsidai, Ayoosh Pareek, Eric Hamrin Senorski, Alberto Grassi, Christophe Ley, Umile Giuseppe Longo, Elmar Herbst, Michael T. Hirschmann, Sebastian Kopf, Romain Seil, Thomas Tischer, Kristian Samuelsson and Robert Feldt. All authors have read the final manuscript and given final approval of the manuscript to be published. Each author consented to be accountable for all aspects of the research in ensuring that questions related to the accuracy or integrity of any part of the work are appropriately investigated and resolved.

## CONFLICT OF INTEREST STATEMENT

Michael T. Hirschmann is a consultant for Medacta, Symbios and Depuy Synthes. Kristian Samuelsson is a member on the board of directors for Getinge AB (publ). Robert Feldt is Chief Technology Officer and founder in Accelerandium AB, a software consultancy company.

## ETHICS STATEMENT

The authors have nothing to report.

## Data Availability

Data sharing not applicable to this article as no data sets were generated or analysed during the current study.
